# Low-Temperature Treatment of Boehmitic Bauxite Using the Bayer Reductive Method with the Formation of High-Iron Magnetite Concentrate

**DOI:** 10.3390/ma16134678

**Published:** 2023-06-28

**Authors:** Andrei Shoppert, Dmitry Valeev, Irina Loginova, Denis Pankratov

**Affiliations:** 1 Department of Non-Ferrous Metals Metallurgy, Ural Federal University, 620002 Yekaterinburg, Russia; i.v.loginova@urfu.ru; 2 Laboratory of Advanced Technologies in Non-Ferrous and Ferrous Metals Raw Materials Processing, Ural Federal University, 620002 Yekaterinburg, Russia; 3 Laboratory of Sorption Methods, Vernadsky Institute of Geochemistry and Analytical Chemistry, Russian Academy of Sciences, 119991 Moscow, Russia; dmvaleev@yandex.ru; 4 Department of Chemistry, Lomonosov Moscow State University, 119991 Moscow, Russia; pankratov@radio.chem.msu.ru

**Keywords:** boehmite, atmospheric leaching, alkali, hematite reduction, red mud valorization, Mössbauer spectroscopy

## Abstract

The Bayer process is the main method of alumina production worldwide. The use of low-quality bauxites for alumina production results in the formation of a significant amount of technogenic waste—bauxite residue (BR). The Bayer reductive method is one possible way to eliminate BR stockpiling, but it requires high-pressure leaching at temperatures higher than 220 °C. In this research, the possibility of boehmitic bauxite atmospheric pressure leaching at both the first and second stages or high-pressure leaching at the second stage with the simultaneous reduction of hematite to magnetite was investigated. Bauxite and solid residue after NaOH leaching were characterized using XRD, SEM-EDS, and Mössbauer spectroscopy methods. The first stage of leaching under atmospheric pressure with the addition of Fe(II) species in a strong alkali solution (330–400 g L^–1^ Na_2_O) resulted in a partial reduction of the iron minerals and an extraction of more than 60% of Si and 5–25% of Al (depending on caustic modulus of solution) after 1 h. The obtained desilicated bauxite was subjected to atmospheric leaching at 120 °C in a strong alkali solution (350 g L^−1^) or high-pressure leaching at 160–220 °C using the Bayer process mother liquor in order to obtain a concentrate with a magnetite content higher than 83 wt. %.

## 1. Introduction

Depending on the composition and properties of the feedstock, alumina (Al_2_O_3_) is produced by a variety of industrial processes [1]. Low-silica bauxites are used worldwide for the production of alumina by the most energy-effective Bayer process, which is based on alkaline leaching with continuous regeneration of the solution by the desilication and precipitation of aluminum hydroxide (Al(OH)_3_) [2]. However, lower quality raw materials with a silica modulus (η_Si_, Al_2_O_3_ to SiO_2_ mass ratio) lower than seven such as high-silica bauxites, kaolin, clays, argillites, alkaline aluminosilicates, coal ash, coal gangue, aluminum dross, and other materials will be used in the near future. The use of the Bayer method for the treatment of such materials becomes ineffective because of the formation of a high amount of the desilication product (DSP, Na_6_[Al_6_Si_6_O_24_]·Na_2_X, where Na_2_X is inorganic compounds of Na) [1].

Sintering with soda (Na_2_CO_3_) and lime (CaO) [3,4] acid methods [5,6] was developed for low-grade ores. However, almost all the alumina from such raw materials is currently produced by sintering and combined alkaline sintering processes [7], which are high energy-intensive and produce a large amount of solid waste (red mud or BR).

This is why the following modified alkaline methods based on the Bayer process are being extensively developed:

(a) Gravity separation followed by the Bayer process [8,9];

(b) Aluminosilicate flotation followed by the Bayer process [10,11,12];

(c) Chemical pre-desilication followed by the Bayer process [13,14];

(d) The reductive Bayer process [15,16].

The reductive Bayer process is the most promising method from the perspective of BR elimination because it makes it possible to convert BR in the easy to process by-product with a high iron content in the form of magnetite (Fe_3_O_4_) [17,18]. The mechanism of the magnetite formation can be described by Equations (1)–(3). The source of the Fe^2+^ can be different: iron [19] and aluminum [20] that reacts with the alkaline solution with the formation of H_2_ and following the reduction of Fe^3+^ to Fe^2+^; the addition of FeSO_4_ [21,22] and the addition of organic matter [23]. The presence of Fe^2+^ ions also helps to decrease the amount of Na and Al that is lost with the BR, which is connected to the formation of other desilication products [24]. However, the main disadvantage of these methods is the need of a high-pressure leaching process at T > 220 °C.

Fe + H_2_O + OH^−^ = HFeO_2_^−^ + H_2_, (1)

Fe^2+^ + 3OH^−^ = HFeO_2_^−^ + H_2_O, (2)

Fe_2_O_3_ + HFeO_2_^−^ = Fe_3_O_4_ + OH^−^. (3)

In a recent work by Li et al. [23], it was shown that the use of a two-stage process, where in the first stage, the major part of the Si-containing materials from gibbsitic bauxite was transferred to the solution, and the magnetization of goethite (𝛾-FeOOH) was accomplished at the second stage at 270 °C, resulted in the complete dissolution of Al and the formation of the BR, which could be used as a substituent for iron concentrates.

In our previous research, it was shown that there is a possibility of performing the complete magnetization of 𝛾-FeOOH at atmospheric pressure by using a highly concentrated alkaline solution (C_Na2O_ > 330 g L^–1^) [25]. In this research, this process was used for the magnetization of hematite (Fe_2_O_3_), which is a part of high-silica and high-iron boehmitic bauxite. To eliminate DSP formation, the process was accomplished in two stages, where in the first stage, the desilication of the bauxite was made in the presence of Fe^2+^, which begins to react with the iron minerals. In the second stage, the desilicated bauxite was subjected to atmospheric leaching at 120 °C in a strong alkali solution or high-pressure leaching at 160–220 °C in the Bayer process mother liquor to obtain a magnetite concentrate with an iron content higher than 58%. The bauxite and solid residues were characterized using Mössbauer spectroscopy, X-ray diffraction, and SEM-EDS analyses to reveal the transformation of iron minerals to magnetite.

## 2. Materials and Methods

### 2.1. Materials

The raw bauxite was obtained from the RUSAL-Kamensk-Uralsky alumina refinery (56.304530, 61.980334; Kamensk-Uralsky, Russia). The refinery uses high-silica boehmitic bauxite from the Timan Deposit (Uchta, Russia) for alumina production using the Bayer sintering process.

Alkaline solutions were prepared by dissolution of a pre-determined amount of solid NaOH (JSC Soda, Sterlitamak, Russia) in 300 mL of distilled water. When fully dissolved, the volume was adjusted with water to obtain a solution with a Na_2_O concentration of 330, 360, or 400 g L^−1^ (C_Na2O_). Solutions with different initial concentrations of 190 and 380 g L^−1^ Al_2_O_3_ (C_Al2O3_) were prepared by dissolving Al(OH)_3_ (JSC BaselCement-Pikalevo, Pikalevo, Russia) in a hot alkaline solution in order to study the effect of the Al concentration in the solution on the leaching process.

### 2.2. Experimental

Pre-desilication with a caustic alkali solution and desilicated bauxite leaching were carried out in a thermostated 0.5 L stainless steel reactor and in high-pressure reactors, respectively. The reactor had openings for the loading of chemical reagents as well as for temperature control and the recirculation of evaporated water through a water-cooled condenser. The high-pressure reactors were hermetically sealed steel vessels placed in an air thermostat with mixing through the head. The stirring speed in all experiments was 700 rpm for the reactor and 40 rpm for the high-pressure reactors because the leaching efficiency does not increase at higher speeds [26]. Crushed ore and the required amount of lime (analytical purity) were added to a solution with a Na_2_O concentration of 330, 360, or 400 g L^–1^ and an initial concentration of Al_2_O_3_ of 0, 190, and 380 g L^–1^ on the basis of obtaining the required L:S ratio (mL g^–1^). For the simultaneous process of hematite magnetization, in addition to bauxite, the stoichiometric (relative to the trivalent iron in the magnetite or in accordance with Equation (3)) amount of divalent iron as FeSO_4_-7H_2_O (analytical purity) was added. After leaching, the pulp was filtered; the solid residue was dried at 110 °C for 240 min before analysis.

### 2.3. Methods of Analysis

The mineralogy of the raw bauxite and the solid residue after alkaline leaching was measured by X-ray diffraction (XRD) using a Difrei-401 X-ray diffractometer (JSC Scientific Instruments, Saint Petersburg, Russia) with a Cr-Kα radiation source and a 2θ range of 14° to 140° with an exposure of 30 min. The x-ray source operating mode was set to 25 kW/4 mA. Mineral phase analysis was performed with Match! 3 software (Crystal Impact, Bonn, Germany).

An Axios Max X-ray fluorescence (XRF) spectrometer (Panalytical, Almelo, The Netherlands) was used to analyse the solid residue after pulp filtration. Scanning electron microscopy with energy dispersive X-ray spectroscopy (SEM-EDX, Vega III, Tescan, Brno, Czech Republic) was used to study the surface morphology and elemental composition of the raw bauxite and solid residues. 

Particle size distributions of the raw bauxite and the solid residues were examined via an Analysette 22 NanoTec (Fritsch, Idar-Oberstein, Germany).

The ^57^Fe Mössbauer absorption spectra were obtained on an express Mössbauer spectrometer MS1104EM (CJSC Kordon, Rostov-on-Don, Russia) at temperatures of 296 ± 3 and 77.7 ± 0.3 K. In this case, the source of γ-radiation with an activity of 40 mCu in the form of ^57^Co/Rh (Cyclotron Co., Ltd., Obninsk, Russia) was at room temperature. The noise/signal ratio for the spectra did not exceed 2%. Mathematical processing of the experimental Mössbauer spectra was carried out for the high-resolution spectra (1024 points) using the SpectRelax 2.8 program (Lomonosov Moscow State University, Russia). The isomeric shifts values are given relative to α-Fe.

### 2.4. Calculations

The amount of elements extracted from the bauxite (X) was calculated using Equation (4):

X = (m_1_ × X_1_ − m_2_ × X_2_)/(m_1_ × X_1_), (4)

where m_1_ is the original sample amount (g); X_1_ is the element content in the raw bauxite (%); m_2_ is the leaching residue amount (g); X_2_ is the element content in the solid residue (%).

Statistica 13 software (TIBCO, Hamburg, Germany) was used for experimental planning that helps to avoid interaction between factors and to reduce the number of experiments. The design is made up of three blocks of sixteen experiments each, with the parameters being varied at three levels. The output parameters were the Al and Si extraction and the content of Fe and Na in the leaching residue. 

To create a model of the Al and Si extraction and the Fe and Na content in the leaching residue as a function of the variables, a statistical automated neural network (SANN) was used. SANN is an artificial intelligence based method. It uses learning methods to adjust the modelling result until the desired quality is achieved. For the study of the bauxite leaching process, a multilayer perceptron (MLP) method was used.

## 3. Results and Discussion

### 3.1. Raw Bauxite Characterization

Raw bauxite from the Timan Deposit was pre-crushed using a rod mill and subsequently classified on vibrating sieves (NKP Mekhanobr-Tekhnika, Saint-Petersburg, Russia) to achieve a particle size of 80% less than 71 μm. The crushed bauxite before the experiments was subjected to the sieve procedure to obtain three fractions: −50 μm, +50–71 μm, and +71 μm. The average particle size of each fraction was: 48 μm, 62 μm, and 87 μm. The chemical composition of these three fractions and the raw bauxite is shown in Table 1.

According to the data presented in Table 1, the raw bauxite is high-iron and highly siliceous. The silica modulus (the ratio of Al_2_O_3_ to SiO_2_) of bauxite is 8.36 units, which is at the lower limit of profitability for Bayer’s method.

**Figure 1:**
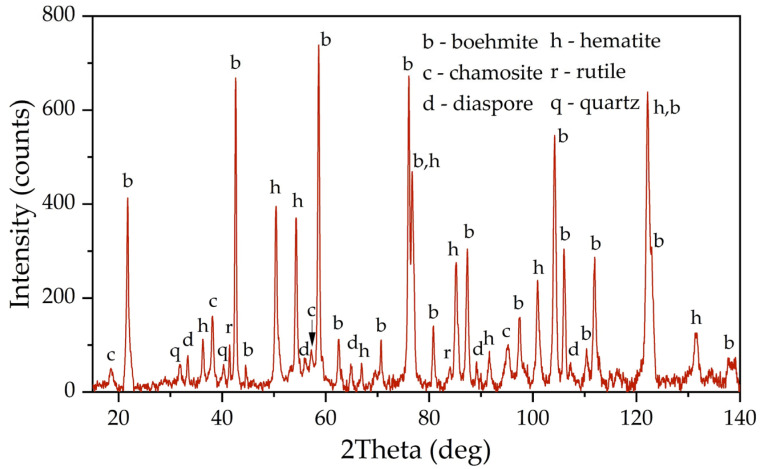
XRD pattern of the raw bauxite of the Timan Deposit.

Figure 1 shows an X-ray diagram of the raw bauxite. Raw bauxite consists mainly of boehmite (AlOOH) and hematite (Fe_2_O_3_). Small amounts of rutile (TiO_2_), quartz (SiO_2_), diaspore (AlOOH), chamosite ((Fe^2^⁺,Mg,Al,Fe^3^⁺)_6_(Si,Al)_4_O_10_(OH,O)_8_) are also present. A semi-quantitative analysis of the crystalline phases of the bauxite sample is shown in Table 2. According to Table 2, more than 62% of the original bauxite is represented by boehmite, more than 25% by hematite, the rest is quartz, rutile, and chamosite. However, it should be noted that chamosite also has in its composition both alumina and silica, which may lead to subsequent problems during leaching (secondary aluminum losses due to the formation of DSP). According to the literature [6], kaolinite is often found in high-silica bauxite, but its content in this sample of Timan bauxite was insignificant.

**Figure 2:**
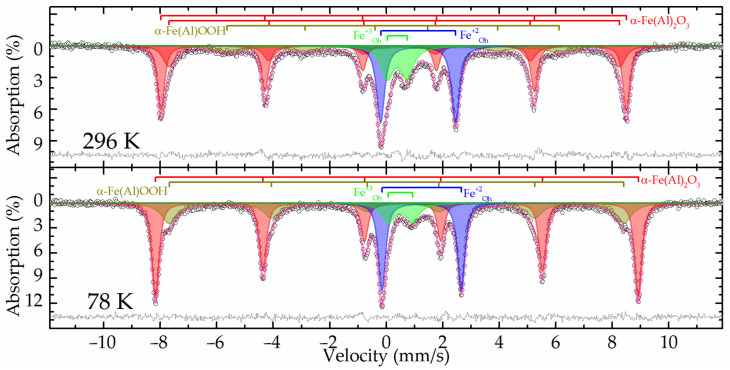
Experimental Mössbauer spectra and the models for their description for the raw bauxite sample.

The Mössbauer spectra at both temperatures of the raw bauxite sample showed a set of rather narrow resonance lines in which the presence of a sextet and a doublet with a large quadrupole splitting was clearly distinguished (Figure 2). The experimental spectra could be satisfactorily described by a superposition of four or five subspectra including two symmetrical doublets and two or three symmetrical sextets (Table 3).

In the spectrum obtained at room temperature, two sextets with the maximum values of hyperfine magnetic splitting (Table 3, subspectra 1 and 2) corresponded to hematite—α-Fe_2_O_3_ as well as aluminum-substituted hematite [25]. When the sample was cooled to the boiling point of nitrogen, these two sextets combined into one sextet (Figure 2). In this case, the value of the quadrupole shift did not change sign, which indicates the absence of the Morrin transition characteristic of pure hematite, and confirms the hypothesis of aluminohematite formation [27]. The remaining sextet demonstrated a strong temperature dependence of both its profile and the hyperfine magnetic splitting (Table 3, subspectrum 3). The hyperfine parameters of this subspectrum and the features of its temperature changes allow it to be attributed to aluminogoethite, which we considered in detail in [5]. The rest of the spectrum can be described by a pair of doublets corresponding to iron atoms with charges of +3 and +2 (Table 3, subspectra 4 and 5) in the high-spin state and octahedral oxygen environment [28]. Considering that the intensity of subspectrum 4 (Table 3) decreases almost twofold with decreasing temperature, it can be assumed that superparamagnetic aluminogoethite is partly responsible for the formation of this subspectrum. The rest of this subspectrum as well as subspectrum 5 obviously belonged to a layered aluminosilicate mineral, in particular, the hyperfine parameters made it possible to reliably assign them to chamosite [29,30,31].

**Figure 3:**
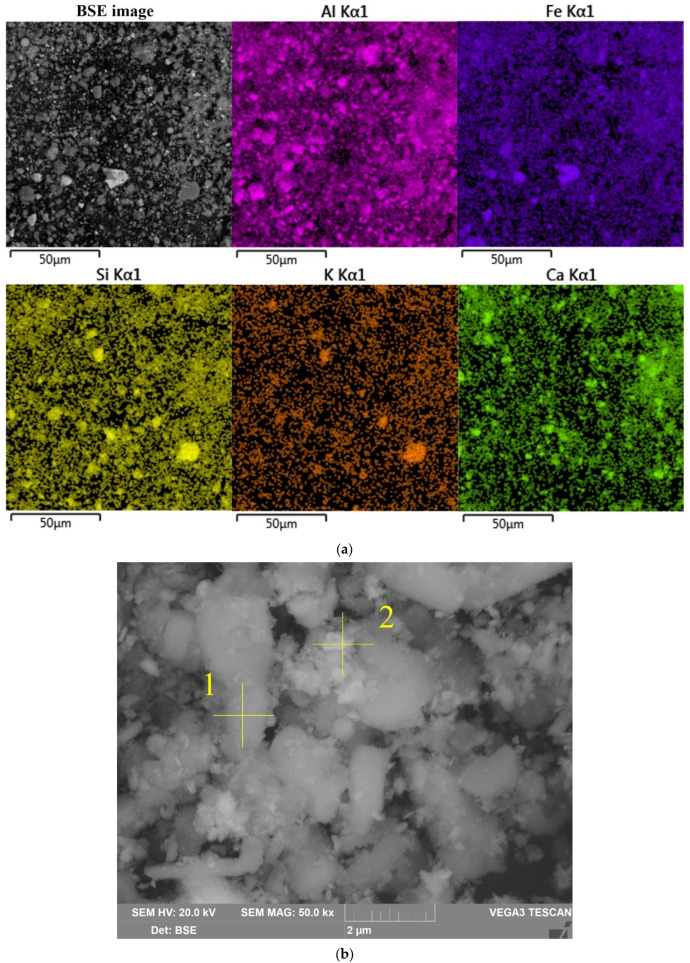
Results of the bauxite surface elemental analysis by SEM-EDS (**a**). Bauxite particles with EDS spectra (yellow cross) position (**b**).

The morphology and chemical composition of the raw bauxite particles were evaluated by SEM-EDS analysis (Figure 3, Table 4). The SEM-EDS images in Figure 3 show that aluminum, iron, silicon, and calcium were uniformly distributed over the surface of the bauxite particles, but single particles with a high content of these elements could be identified. Potassium has a close association with silica, indicating its content in the aluminosilicates.

Figure 3b shows that the particles of raw bauxite had an irregular shape. After grinding, it was possible to observe particles with sizes from 100 nm to 10 μm. SEM-EDS image of the boehmite and hematite particles are shown in Table 4. There was a close relationship between them (i.e., the boehmite particles were covered with hematite particles and vice versa).

### 3.2. Effect of Leaching Conditions on Bauxite Desilication and Transformation of Iron Minerals on the First Stage

Modeling of the process of bauxite pretreatment with highly concentrated alkaline solutions was carried out using the SANN model.

As revealed in a previous study [25], the use of high alkali concentrations and the L:S ratio eliminates the formation of DSP due to silicon retention in the solution. This allows for a complete extraction of alumina, even from such highly silica raw materials such as fly ash, regardless of how much silica was contained in the feedstock. Moreover, the boiling temperature of the highly concentrated NaOH solution (more than 330 g L^−1^ Na_2_O) exceeded 120 °C. This makes leaching at atmospheric pressure possible at temperatures above 100 °C. The matrix of experiments created with the Statistica 13 software package and the results of Al and Si extraction from the different bauxite fractions and the Fe and Na_2_O content in the solid residue are shown in Table 5.

As was shown [32,33], the machine learning produces more accurate models than the use of convenient methods. The closest to the experimental data SANN model (R^2^ = 0.96) obtained for alumina extraction was multilayer perceptron (MLP) 6.10.4, where 6 was the number of input parameters, 10 was the number of hidden layers, and 4 the number of output layers.

**Figure 4:**
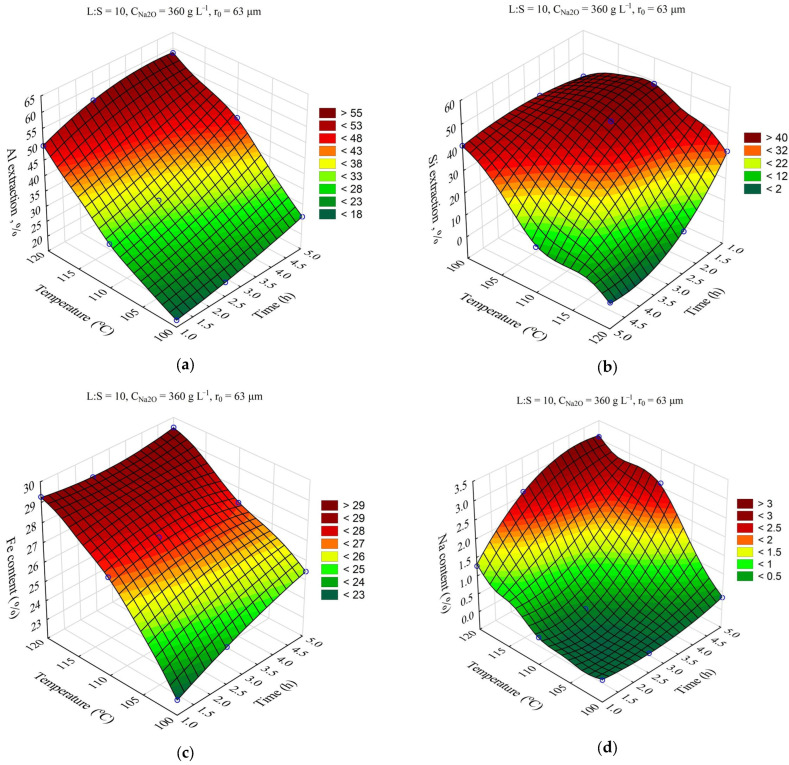
Response surfaces for the effect of time and temperature on: Al extraction (**a**); Si extraction (**b**); Fe content in the solid residue (**c**); Na content in the solid residue (**d**).

The response surfaces predicted by the SANN model for the effect of time and temperature on the degree of Al and Si extraction and the Fe and Na content in the solid residue are shown in Figure 4. The leaching time (τ, min) was varied from 1 to 5 h, and the temperature (T, °C) from 100 to 120 °C. The L:S ratio, Na_2_O concentration (C_Na2O_, g L^–1^), initial Al_2_O_3_ concentration (C_Al2O3_, g L^–1^), and initial mean particle size (r_0_, μm) were fixed at L:S = 10, r_0_ = 63 μm, C_Na2O_ = 360 g L^–1^, and C_Al2O3_ = 0 g L^–1^.

Obviously, increasing the temperature and time allowed for an increase in the Al and Si extraction up to 60 and 40–50%, respectively. However, the effect of time on the Fe content in the solid residue was low, especially at high temperature. This may have been due to the fact that the desilication was completed in the first hour. Then, according to Figure 4d, the DSP began to precipitate, which led to an increase in the Na_2_O content in the precipitate up to 3.2%, and accordingly, it led to a decrease in the Fe content.

**Figure 5:**
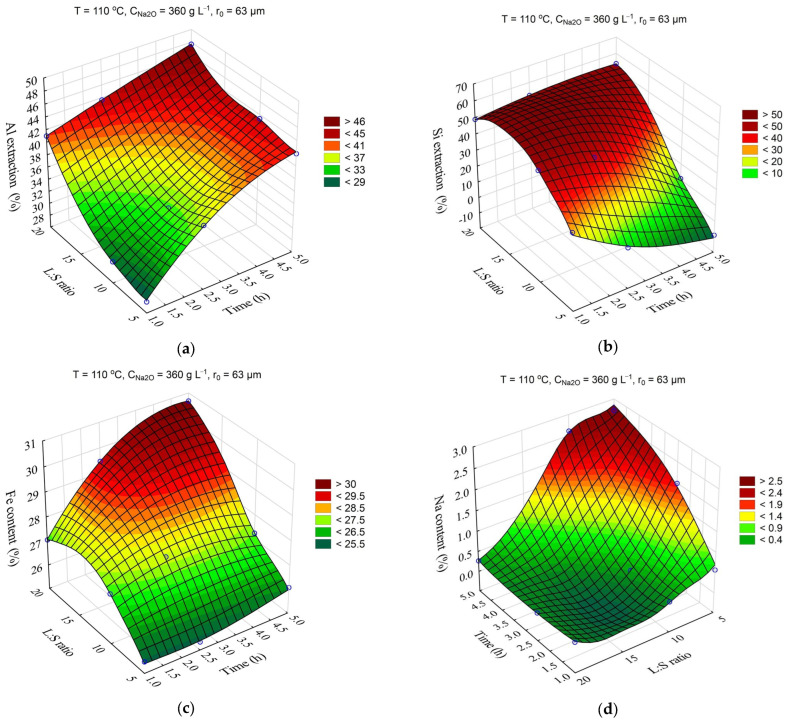
Response surfaces for the effect of time and L:S ratio on: Al extraction (**a**); Si extraction (**b**); Fe content in the solid residue (**c**); Na content in the solid residue (**d**).

The response surfaces predicted by the SANN model for the effect of the time and L:S ratio on the Al and Si extraction as well as the Fe and Na content in the solid residue are shown in Figure 5. The leaching duration (τ, h) was varied from 1 to 5 h, and the ratio L:S from 5 to 20. Other parameters were fixed at T = 110 °C, r_0_ = 63 µm, C_Na2O_ = 360 g L^–1^, and C_Al2O3_ = 0 g L^–1^.

The increase in L:S from 5 to 20 allowed to increase the solutional extraction from 28 to 41% after 1 h of leaching (Figure 5a). After 5 h of leaching, the increase in L:S from 5 to 20 resulted in an increase in the Al extraction by only 6%. At the same time, the increase in L:S allowed for a significant increase in the Si extraction (Figure 5b), which was associated with its retention in the solution, as evidenced by the Na content in the solid residue, which increased to 3% after 5 h at L:S = 5. The high Al and Si extraction at L:S above 10 also led to an increased Fe content in the solid residue, since Fe is not leached out during the alkaline treatment and concentrates in the residue.

**Figure 6:**
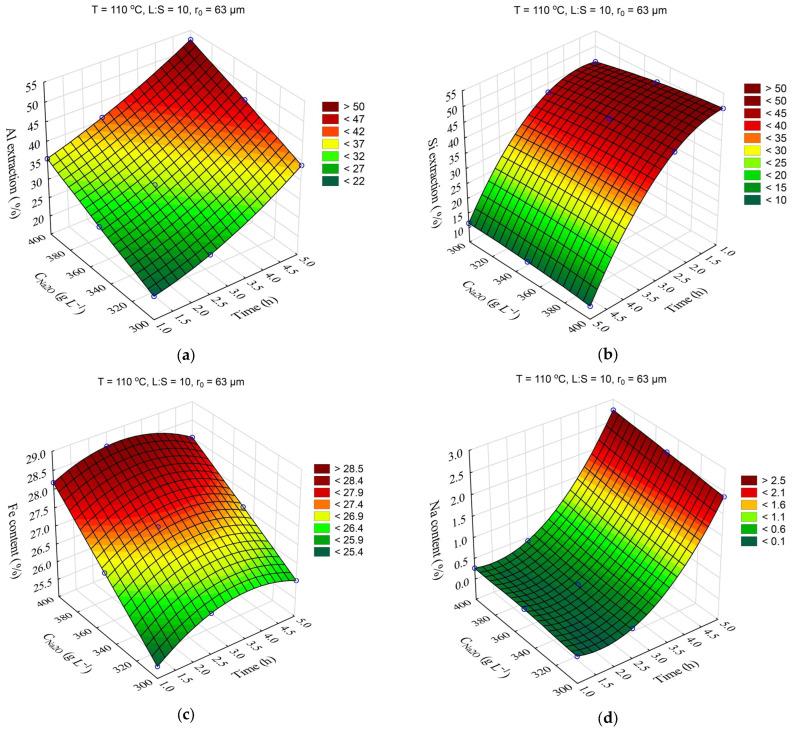
Response surfaces for the effect of time and Na_2_O concentration in the solution on: Al extraction (**a**); Si extraction (**b**); Fe content in the solid residue (**c**); Na content in the solid residue (**d**).

The response surfaces predicted by the SANN model for the effects of time and Na_2_O concentration on the Al and Si extraction and the Fe and Na content in the solid residue are shown in Figure 6. The leaching time (τ, min) was varied from 1 to 5 h, and the Na_2_O concentration from 330 to 400 g L^–1^. The other parameters were fixed at T = 110 °C, r_0_ = 63 µm, L:S = 10, and C_Al2O3_ = 0 g L^–1^.

The data in Figure 6a show that the solution composition had a significant impact on the Al extraction, which seems to be associated with an increase in the caustic modulus and as a consequence, with the increased equilibrium concentration of Al in solution. Thus, an increase in Na_2_O concentration from 330 to 400 g L^–1^ after 5 h of leaching led to an increase in Al extraction from 40 to 54%. The effect of the solution concentration on the Si extraction and Na_2_O content in the solid residue was insignificant (Figure 6b,d). Increased Al extraction at a high concentration reduced the yield of the solid residue and, accordingly, increased the iron content. As the leaching duration increased from 3 h to 5 h, DSP began to form, resulting in an increased yield and higher Na_2_O content in the solid residue (Figure 6c,d).

**Figure 7:**
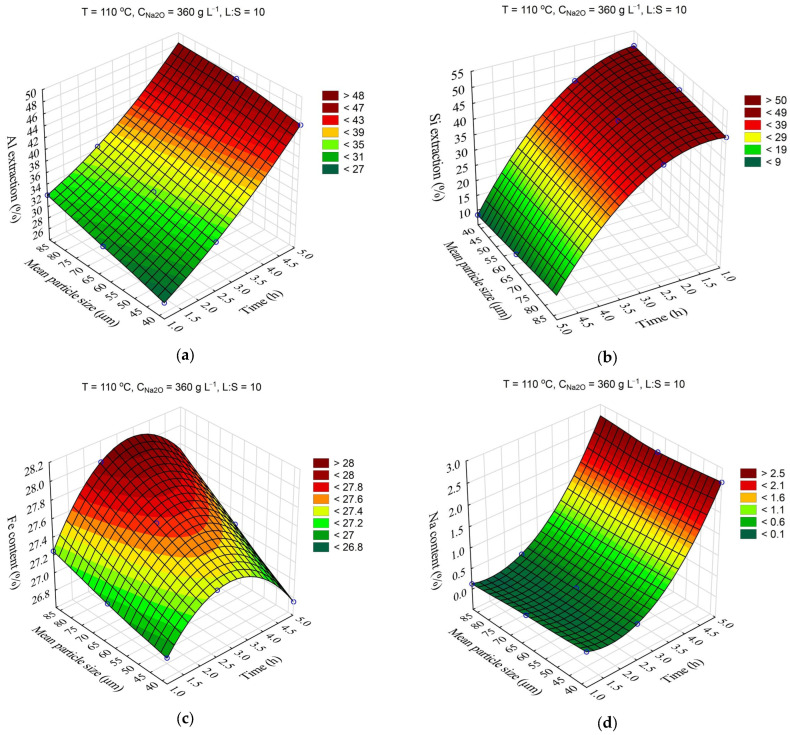
Response surfaces for the effect of time and the initial median particle size (r_0_) on: Al extraction (**a**); Si extraction (**b**); Fe content in the solid residue (**c**); Na content in the solid residue (**d**).

The response surfaces predicted by the SANN model for the effects of time and the initial mean particle size (r_0_) on the Al and Si extraction and the Fe and Na content in the solid residue are shown in Figure 7. The leaching time was varied from 1 to 5 h, and the mean particle size from 38 to 78 μm. Other parameters were fixed at T = 110 °C, C_Na2O_ = 360 g L^–1^, L:S = 10, and C_Al2O3_ = 0 g L^–1^.

**Figure 8:**
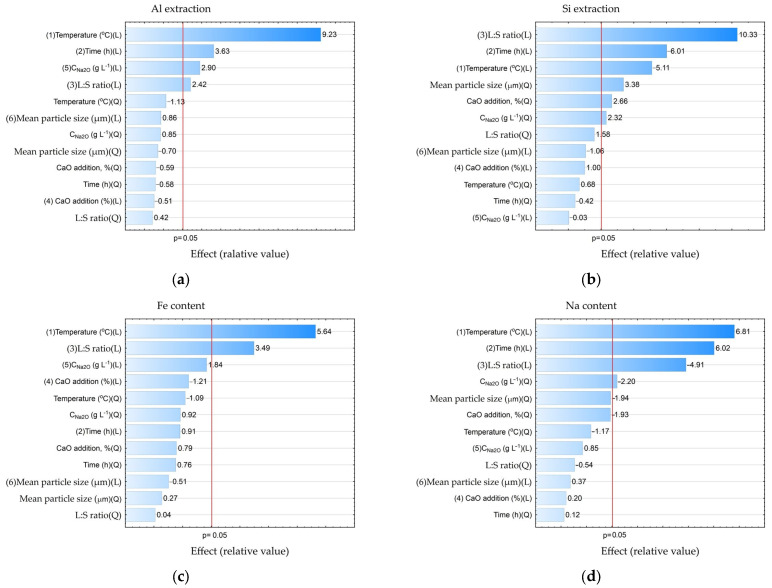
Pareto charts obtained using ANOVA analysis for: Al extraction (**a**); Si extraction (**b**); Fe content in the solid residue (**c**); Na content in the solid residue (**d**).

A decrease in the average particle size from 78 to 38 μm resulted in only a slight (2–4%) increase in Al and Si extraction (Figure 7a,b). The Fe content also slightly increased with the decrease in the r_0_ (Figure 8c), which was associated with a higher Al and Si extraction. After 2.5 h of leaching, the Fe content began to decrease, which is connected to the beginning of DSP formation (Figure 7d).

The data presented in Table 4 were further processed in Statistica using the ANOVA (analysis of variance) method to study the statistical significance of certain process parameters. Pareto diagrams for each variable were constructed based on the results of the ANOVA (Figure 8).

According to the results shown in Figure 8 with a 0.95 confidence level (or significance level of 0.05), the temperature, time, concentration, and L:S ratio were statistically significant for Al and Si extraction: L:S ratio, time (negative effect), temperature (negative effect), average particle size (Q—quadratic dependence); for Na in solid residue, temperature and duration were significant, while the L:S ratio was significant for Na reduction in solid residue. Thus, if the task of the first stage is a selective Si extraction with minimization of Al extraction, it is necessary to take the minimum values of temperature and time and the maximum value L:S. The other parameters were of little importance for Al and Si extraction. Accordingly, the recommended parameters can be seen as follows: T = 100 °C, τ = 1 h, and L:S = 20. Using these parameters, it is possible to extract up to 60% of Si, and the Al extraction can be as low as 20–24%.

**Figure 9:**
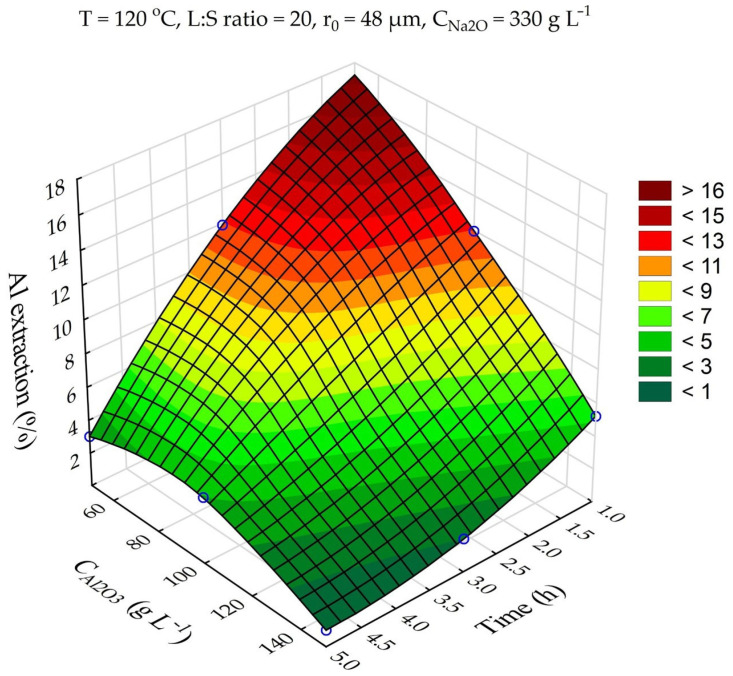
Influence of leaching time and Al_2_O_3_ concentration in solution on Al extraction in the first stage.

Experiments on desilication with the use of aluminate solutions of different Al concentrations were carried out to investigate the possibility of reducing Al co-extraction. It is known that the solubility of boehmite at atmospheric pressure is very low [34], but when using highly concentrated NaOH solutions, it is sufficient to extract more than 50% of aluminum at a L:S above 10. The effect of Al concentration (in terms of Al_2_O_3_, g L^–1^) on the Al extraction from bauxite during the first stage of leaching are shown in Figure 9.

It is obvious that the use of an aluminate solution can suppress the process of Al co-extraction from bauxite during its desilication, even under the most severe conditions: T = 120 °C, Na_2_O = 330 g L^–1^, and the L:S ratio = 20. When the aluminate solution used for desilication at 100 °C, τ = 1 h, and L:S ratio = 20, the Al and Si extraction were 5.1% and 60.5%, respectively. The chemical composition of the concentrate (desilicated bauxite—DB) obtained under these conditions is shown in Table 6. As can be seen, the silica modulus of bauxite after desilication increased to 21.34 units compared with 8.36 units for the original bauxite. The maximum theoretical Al extraction (maximal Al extraction minus Al that will be precipitated as DSP) from this bauxite by the Bayer method is 95.3%.

### 3.3. The Effect of Leaching Conditions on Al Extraction from Desilicated Bauxite (DB)

**Figure 10:**
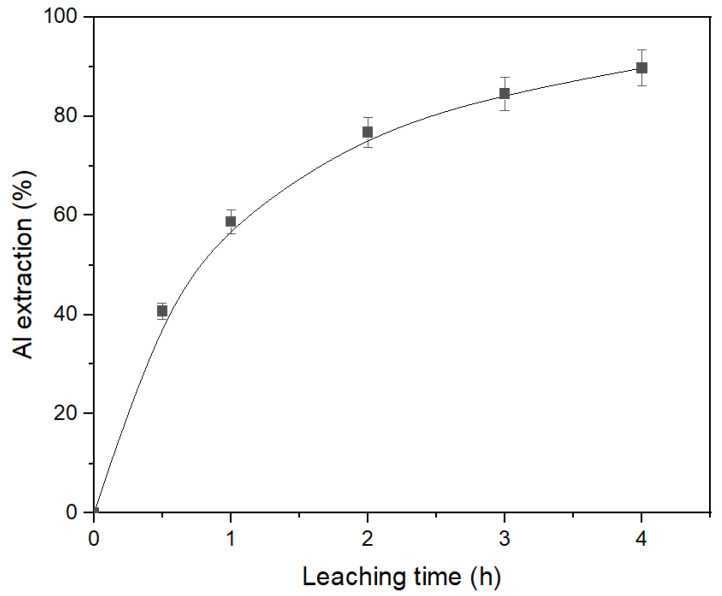
Results of the atmospheric leaching of desilicated bauxite (DB) at T = 120 °C, C_Al2O3_ = 0 g L^−1^, C_Na2O_ = 360 g L^–1^, and the L:S ratio = 20.

The desilicated bauxite obtained in Section 3.1, Table 5 was subjected to second stage leaching under atmospheric pressure. The parameters of the leaching were: T = 120 °C, C_Na2O_ = 360 g L^–1^, C_Al2O3_ = 0 g L^−1^, and the L:S ratio = 20. The result of the effect of time on Al extraction under these condition are shown in Figure 10. As can be seen, DB can be efficiently treated using the atmospheric leaching process. After leaching, the hematite transformation to magnetite was completed of 75.6%. However, the leaching time should be more than 4 h for the extraction of 90% of Al. The resulting aluminate solution contained only 32.6 g L^−1^ Al_2_O_3_ and could not be processed using the Bayer process. Therefore, the high-pressure leaching of DB was studied.

The results of high-pressure Al leaching from DB were also processed using neural network modeling in the Statistica application package. The matrix of experiments and the results of Al extraction from the desilicated bauxite are shown in Table 7.

**Figure 11:**
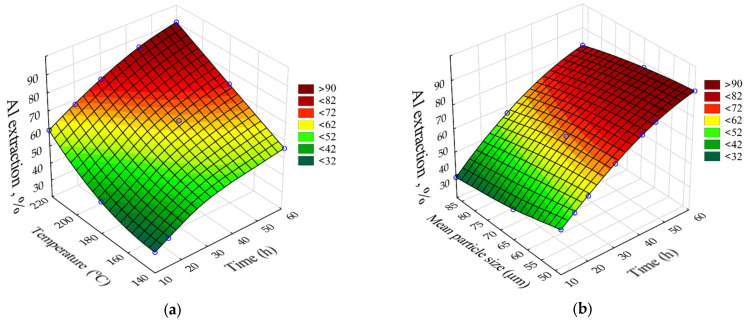
The effect of time and temperature on the Al extraction (**a**) and the effect of duration and initial particle size on the Al extraction from the desilicated bauxite (**b**).

The SANN model that was better fitted to the experimental data of Al extraction was multilayer perceptron (MLP) 5.9.1 (R^2^ = 0.98). The response surfaces predicted by the SANN model for Al recovery as a function of leaching time (τ, h), temperature (T, °C), and initial average bauxite particle size (r_0_, μm) are shown in Figure 11. The fixed values were C_Na2O_ = 330 g L^–1^, C_Al2O3_ = 150 g L^–1^, and the L:S for the obtaining caustic modulus of the aluminate solution α_k_ (molar ratio of Na_2_O to Al_2_O_3_) = 1.65.

The greatest influence (Figure 11) on Al extraction was caused by the leaching time and temperature. Increasing the temperature from 140 °C to 220 °C increased the Al extraction after 60 min of leaching from 56 to 92% (Figure 11a). This may indicate that the surface chemical reaction is the limiting stage of the process. Increasing the initial particle size from 48 μm to 78 μm resulted in a decrease in Al extraction from 90 to 85% (Figure 11b), which may indicate that diffusion has no influence on the kinetics of the leaching process.

### 3.4. Solid Residue Characterization

**Figure 12:**
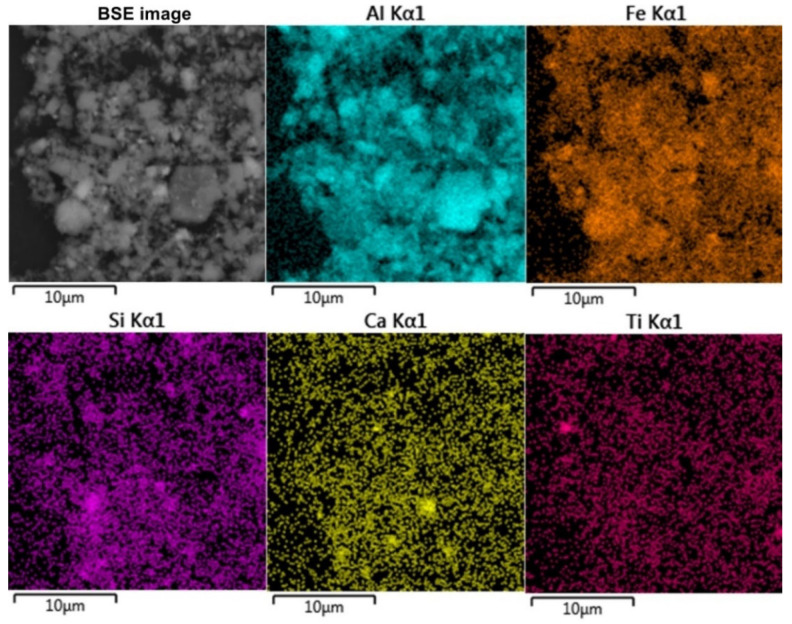
Results of the elemental analysis (SEM-EDS) of the surface of bauxite desilicated at T = 100 °C, C_Na2O_ = 330 g L^–1^ C_Al2O3_ 150 g L^–1^, and L:S ratio = 20.

**Figure 13:**
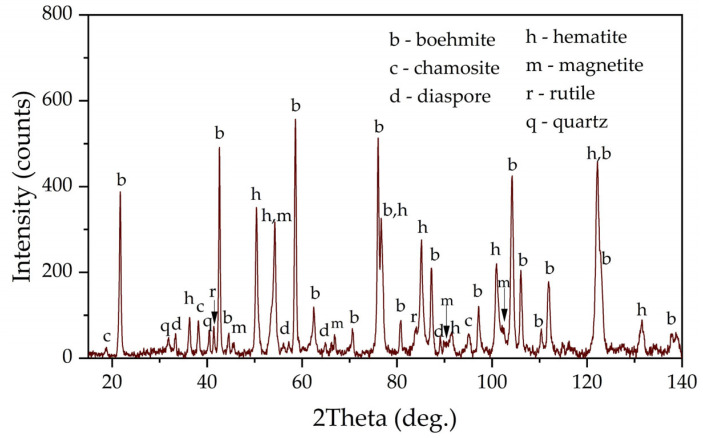
XRD pattern of the desilicated bauxite.

Figure 12 shows the elemental surface distribution maps for the desilicated bauxite after the first stage of leaching at T = 100 °C, C_Na2O_ = 330 g L^–1^ C_Al2O3_ 150 g L^–1^, and L:S ratio = 20. According to the elemental distribution, Fe, Si, Ca, and Ti were evenly distributed over the particle surfaces. Particles of boehmite (particles with high Al content) were clearly visible. Fragmented iron particles were also found, but in general, the iron particles after magnetization appeared to be sufficiently finely dispersed. Figure 13 shows an XRD pattern of bauxite desilicated under the optimum conditions. After desilication in the presence of divalent iron, a new phase, magnetite, appeared, although the intensity of the peaks was low. Furthermore, after desilication and magnetization, the chamosite peaks disappeared, but quartz, which is insoluble at atmospheric pressure, was visible in the solid residue. It should be noted that under the parameters of the Bayer process, chamosite was almost inert up until 200 °C [35].

**Figure 14:**
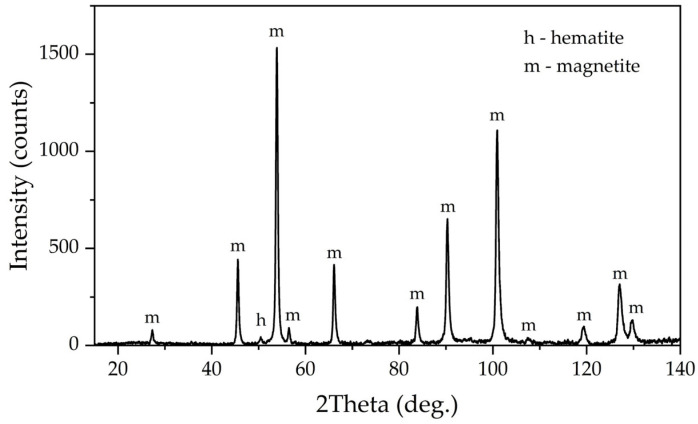
XRD pattern of the bauxite residue obtained by the leaching of desilicated bauxite at T = 220 °C, τ = 120 min, C_Na2O_ = 330 g L^−1^, and C_Al2O3_ = 150 g L^−1^.

The chemical composition of the solid residue obtained after leaching of the DB under conditions similar to the industrial one (T = 220 °C, τ = 120 min, C_Na2O_ = 330 g L^–1^, C_Al2O3_ = 150 g L^–1^, and L:S ratio needed to obtain the caustic modulus of 1.65 in the aluminate solution) is presented in Table 8. The yield of the solid residue was 41.5% from the initial mass of the bauxite sample before desilication. It can be seen that the Fe and Ti content in the residue increased significantly compared to the feedstock, and according to the XRD analysis, almost all iron was represented by magnetite. The alumina content was decreased by a factor of 20, and the silica content by a factor of two. This indicates that practically all of the alumina from the chamosite and all of the boehmite were leached after 2 h of leaching—the total Al extraction after two stages was 97%. At the same time, the Na_2_O content remained very low, even after two stages; this means that the DSP was practically not formed in the leaching of DB in the simultaneous presence of ferrous iron, which was also confirmed by XRD analysis (Figure 14).

XRD pattern in Figure 14 shows that the peaks of boehmite and hematite disappeared, while the peaks of magnetite increased significantly—magnetite remained almost the only phase in this BR. This fact suggests that the leaching of boehmite was complete. However, the absence of DSP and rutile on the XRD means that magnetization also helps to transform both silica and titania in a new phase [36]. The possible chemical reactions of the raw bauxite mineral phases with the leaching agents are listed in Table 9. Fe^2+^ transforms in the alkali media into HFeO_2_^−^, which reacts with the iron and titanium minerals with the formation of magnetite and titanomagnetite. Other minerals dissolve in the solution with the formation of sodium silicate and aluminate without DSP precipitation. Similar XRD patterns of magnetite and titanomagnetite help to explain the absence of the individual mineral of titanium in Figure 14.

**Figure 15:**
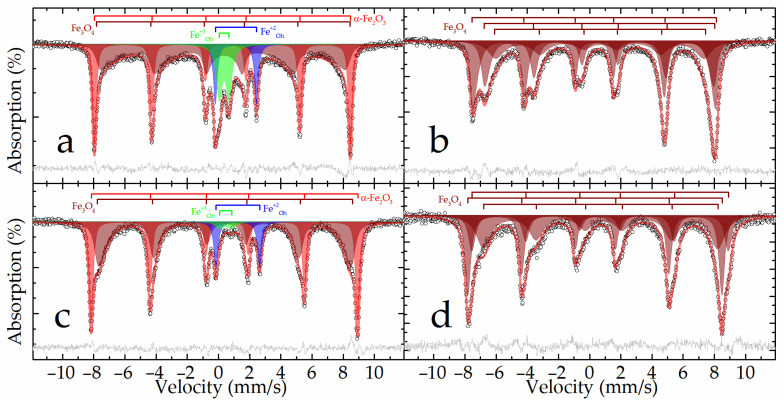
Experimental Mössbauer spectra at 296 (**a**,**b**) and 78 (**c**,**d**) K for the samples DB (**a**,**c**) and bauxite residue (**b**,**d**) and the models for their description.

The results of the XRD patterns in Figure 13 and Figure 14 were confirmed by Mössbauer spectroscopy. The Mössbauer spectra of the DB sample obtained at both temperatures (Figure 15a,c) can be satisfactorily described by the superposition of two sextets and two doublets (Table 3).

The outer sextet with the maximum hyperfine magnetic splitting and narrow resonance lines corresponded to hematite partially substituted by aluminum and was close in parameters to the analogous subspectrum of the raw bauxite sample (Table 3). The intensity of this sextet noticeably increased when going from 296 to 78 K, with a simultaneous decrease in the intensity of the doublet described by subspectrum 3 (Table 3), which suggests that this doublet mainly corresponded to the superparamagnetic nanosized hematite. The hyperfine parameters of subspectrum 4, corresponding to iron(+2) atoms, were similar to the corresponding parameters for the initial bauxite, and obviously corresponded to the incompletely reacted chamosite (Table 3). There were no subspectra that corresponded to goethite in this sample. The rest of the spectrum could only be described using the many-state superparamagnetic relaxation model [37]. The models for the spectra obtained at different temperatures were consistent with each other through the ratio of the energy of the magnetic anisotropy of particles to the thermal energy:

α = K × V/k_B_ × T, (5)

where K—magnetic anisotropy constant; V—volume of the magnetic domain; k_B_—Boltzmann constant; T—temperature [38]. Obviously, this subspectrum refers to the forming particles of nanomagnetite, possibly partially oxidized [39]. From the parameters obtained using Equation (5) and making the assumption that the particles are spherical and that the magnetic anisotropy constant does not depend on temperature and is equal to 2 × 10^4^ J m^−3^ [40,41], one can estimate the size of the magnetic domain for nanomagnetite as 10.32 ± 0.06 nm.

The Mössbauer spectra of the BR sample had a form characteristic of magnetite [42]. There was a sextet with a characteristic splitting of 1–3 resonance lines, an increased intensity of 4–6 lines in the spectra at room temperature, and a noticeable asymmetric distortion of the sextet resonance lines in the spectra at the boiling point of nitrogen (Figure 15b,d). The general broadening of resonance lines to the inner region of the spectrum indicates the manifestation of superparamagnetism by the material [38]. Both spectra were satisfactorily described by the superposition of three sextets. The profile of each was specified within the many-state superparamagnetic relaxation model [37] (Table 3). In this case, within the same spectrum, the sextets were interconnected by relaxation parameters, and the spectra at different temperatures were also consistent with each other through the ratio of the energy of the magnetic anisotropy of particles to the thermal energy (Equation (4)). Similar to the method described above for the example of the DB sample, the sizes of the magnetic domains of nanomagnetite were estimated, which amounted to 19.2 ± 0.2 nm. No other components corresponding to those observed in the raw bauxite or DB samples or not observed in them were recorded in the described spectra, which indicates the complete magnetization of iron minerals after the high-pressure leaching of the DB.

**Figure 16:**
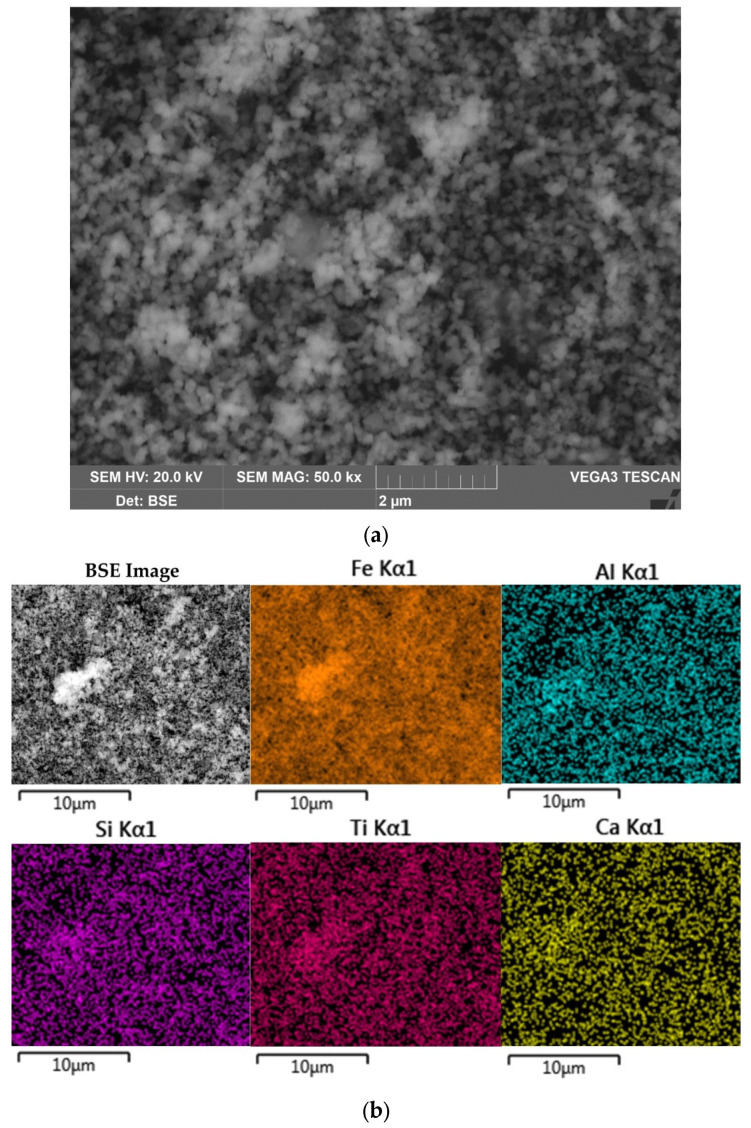
The SEM images of the BR: BSE image of BR at 50,000× magnification (**a**); surface of the BR with elemental distribution maps (**b**).

The morphology and elemental composition of the bauxite residue particles were also investigated using SEM-EDS analysis (Figure 16).

The data obtained above were confirmed by SEM-EDS analysis (Figure 16). Figure 16a,b shows that the particle size of BR (mostly magnetite) was less than 200 nm. At the same time, Al, Si, Ti, and Ca were evenly distributed on the particle surface, which may indicate their inclusion in the iron containing phases. It should be noted that, because of the complete Al extraction and no DSP formation, the obtained BR was enriched in rare-earth elements (REE). For example, the scandium content in BR reached 130 mg kg^−1^. Therefore, the high iron content and concentration of REE make this BR a valuable by-product for the extraction of metals.

## 4. Conclusions

A new method of pre-treating boehmitic bauxite by atmospheric pressure leaching with the addition of Fe^2+^ was examined. According to XRD, Mössbauer spectroscopy, and chemical analysis, Al in the bauxite is mainly represented by boehmite and diaspore, some Al and Si are represented by aluminosilicates—chamosite. The presence of Fe^2+^ facilitates the Al and Si extraction from aluminosilicates (chamosite) and from the solid matrix of iron minerals (Al–goethite and Al–hematite). This effect is due to the magnetization (conversion to magnetite) of hematite and chamosite after their dissolution by an alkaline solution in the presence of Fe^2+^. The results of the Al and Si extraction and Fe and Na content in the solid residue were analyzed using SANN. It was found that the optimum leaching parameters of pretreatment contributing to the maximum Si extraction with minimum aluminum loss were T = 100 °C, τ = 1 h, C_Na2O_ = 330 g L^–1^, C_Al2O3_ = 150 g L^–1^, and L:S ratio = 20. Under these conditions, the Si extraction was higher than 60%, while the Al co-extraction was lower than 10%. After desilication in the presence of ferrous iron, a new phase—magnetite—appeared in the solid residue, according to the X-ray diffraction analysis. According to the SEM-EDS analysis and Mössbauer spectra, the particle size of magnetite was less than 100 nm. The presence of Fe^2+^ during the subsequent leaching of desilicated bauxite using the Bayer process of leaching promoted Al extraction from Al–hematite and chamosite. Additionally, in the presence of Fe^2+^ and low Si content in the feedstock, there was no formation of DSP, which further increased the degree of Al extraction. The optimum leaching parameters for Al extraction from desilicated bauxites were T = 220 °C, τ = 2 h, C_Na2O_ = 330 g L^–1^, and C_Al2O3_ = 150 g L^–1^. Under these conditions, the total Al extraction from the high-iron and high-silica boehmitic bauxite reached more than 97%. The magnetite content in the leaching residue was 83.82%.

## Figures and Tables

**Table 1:** Chemical composition of the Timan bauxite and the three size fractions obtained by the sieve analysis.

Fraction	Main Components, wt. %										
	Al_2_O_3_	Fe_2_O_3_	SiO_2_	CaO	TiO_2_	CO_2_	Na_2_O	MnO	MgO	K_2_O	LOI ^1^
Raw bauxite	52.83	25.90	6.32	0.75	2.70	0.86	0.07	0.51	0.46	0.20	9.62
–50 µm	53.60	25.74	5.76	0.49	2.71	0.86	0.06	0.45	0.42	0.18	9.74
+50–71 µm	53.09	25.84	6.13	0.67	2.71	0.86	0.07	0.49	0.45	0.19	9.51
+71 µm	52.58	25.95	6.51	0.84	2.70	0.86	0.07	0.54	0.47	0.20	9.28

^1^ Lost on ignition at 1000 °C.

**Table 2:** Semi-quantitative mineral composition of the raw bauxite.

Phase	Content (wt.%)
Boehmite	62.3
Hematite	25.7
Rutile	2.6
Quartz	3.6
Chamosite	3.4

**Table 3:** Mössbauer spectral parameters for the samples.

Temperature, K			77.7 ± 0.3						296 ± 3					
Sample	No.	Phase	δ	ε (Δ = 2ε)	Γ_exp_	H_eff_ {H_ext_}	α	S	δ	ε (Δ = 2ε)	Γ_exp_	H_eff_ {H_ext_}	α	S
			mm/s			kOe		%	mm/s			kOe		%
Raw bauxite	1	α-Fe(Al)_2_O_3_	0.48 ± 0.01	−0.10 ± 0.01	0.31 ± 0.01	529.9 ± 0.1		46.0 ± 0.8	0.37 ± 0.01	−0.11 ± 0.01	0.30 ± 0.01	510.8 ± 0.1		32 ± 1
	2								0.38 ± 0.01	−0.10 ± 0.01	0.51 ± 0.02	494.5 ± 0.9		18 ± 1
	3	α-Fe(Al)OOH	0.48 ± 0.01	−0.11 ± 0.01	0.67 ± 0.02	498.6 ± 0.5		24.4 ± 0.9	0.39 ± 0.02	−0.15 ± 0.02	1.31 ± 0.07	365 ± 2		13.7 ± 0.6
	4	Fe^+3^_Oh_	0.51 ± 0.01	(0.86 ± 0.01)	0.55 ± 0.02			8.3 ± 0.3	0.39 ± 0.01	(0.70 ± 0.01)	0.53 ± 0.01			14.0 ± 0.3
	5	Fe^+2^_Oh_	1.25 ± 0.01	(2.81 ± 0.01)	0.31 ± 0.01			21.3 ± 0.2	1.13 ± 0.01	(2.65 ± 0.01)	0.34 ± 0.01			21.9 ± 0.3
DB	1	α-Fe_2_O_3_	0.48 ± 0.01	−0.09 ± 0.01	0.33 ± 0.01	530.0 ± 0.1		38.6 ± 0.7	0.37 ± 0.01	−0.11 ± 0.01	0.31 ± 0.01	509.1 ± 0.1		31 ± 1
	2	Fe_3_O_4_	0.47 ± 0.01	−0.05 ± 0.01	0.52 ± 0.01	508.0 ± 0.4	10.74 ± 0.05	49.0 ± 0.7	0.35 ± 0.01	−0.03 ± 0.01	0.37 ± 0.01	504 ± 3	2.81 ± 0.05	47 ± 1
	3	Fe^+3^_Oh_	0.46 ± 0.02	(0.78 ± 0.04)	0.60 ± 0.01			2.6 ± 0.2	0.37 ± 0.01	(0.61 ± 0.01)	0.46 ± 0.01			12.4 ± 0.2
	4	Fe^+2^_Oh_	1.25 ± 0.01	(2.8 ± 0.01)	0.32 ± 0.01			9.8 ± 0.2	1.13 ± 0.01	(2.64 ± 0.01)	0.29 ± 0.01			9.2 ± 0.2
Bauxite residue	1	Fe_3_O_4_	0.36 ± 0.01	−0.01 ± 0.01	0.42 ± 0.01	505.5 ± 0.1	69.08 ± 0.6	38 ± 1	0.31 ± 0.01	0.00 ± 0.01	0.38 ± 0.01	485.8 ± 0.2	18.3 ± 0.6	42.7 ± 0.6
	2		0.68 ± 0.01	0.00 ± 0.01	0.52 ± 0.02	510.4 ± 0.3		21 ± 1	0.65 ± 0.01	−0.02 ± 0.01	0.47 ± 0.01	458.9 ± 0.3		33 ± 1
	3		0.82 ± 0.01	−0.10 ± 0.01	1.11 ± 0.02	466.2 ± 0.7		41 ± 1	0.69 ± 0.01	−0.02 ± 0.01	1.11 ± 0.03	420 ± 1		25 ± 1

δ—isomeric shift; ε—quadrupole shift; (Δ = 2ε)—quadrupole splitting; Γ_exp_—linewidth; H_eff_—hyperfine magnetic field; α—division of particle anisotropy energy by thermal energy; S—relative subspectrum area.

**Table 4:** Results of the EDS analysis of the raw bauxite (spectra numbers are given in Figure 3), wt.%.

Spectra No.	O	Al	Fe	Si	Ca	Ti	Mn	Phase
1	56.6	27.4	12.9	1.9	1.1	-	-	Boehmite (AlOOH)
2	46.5	14.8	36.5	1.0	0.5	0.4	0.4	Hematite (Fe_2_O_3_)

**Table 5:** Matrix for experiments on the desilication of Middle Timan bauxite at atmospheric pressure.

Time (h)	Temperature (°C)	L:S Ratio	r_0_ (µm)	C_Na2O_ (g L^–1^)	CaO Addition (%)	Al Extraction (%)	Si Extraction (%)	Fe Content (%)	Na Content %)
1.0	100	10	63	360	0	20.95	42.69	24.23	0.16
1.0	120	10	63	360	0	31.90	33.10	26.52	0.24
5.0	100	10	63	360	0	24.97	40.80	24.91	0.24
5.0	120	10	63	360	0	51.05	0.00	29.49	2.03
1.0	100	10	63	360	6	23.35	46.36	23.97	0.16
1.0	120	10	63	360	6	73.80	40.77	35.26	0.92
5.0	100	10	63	360	6	26.42	44.71	24.50	0.28
5.0	120	10	63	360	6	62.56	0.00	28.03	4.70
1.0	110	5	63	330	3	13.94	27.34	23.24	0.47
5.0	110	5	63	330	3	36.78	0.00	25.54	2.03
1.0	110	20	63	330	3	28.78	57.70	25.43	0.086
5.0	110	20	63	330	3	39.67	58.72	27.63	0.13
1.0	110	5	63	400	3	33.93	24.98	25.50	0.76
5.0	110	5	63	400	3	46.64	0.00	26.57	2.53
1.0	110	20	63	400	3	24.27	63.46	27.39	0.16
5.0	110	20	63	400	3	58.04	58.55	32.32	0.40
2.5	110	5	38	360	0	25.16	0.00	23.90	1.65
2.5	110	20	38	360	0	36.90	50.14	27.81	0.23
2.5	110	5	38	360	6	30.39	9.95	23.85	1.34
2.5	110	20	38	360	6	32.20	45.89	27.92	0.26
2.5	110	5	87	360	0	59.36	2.00	33.53	2.50
2.5	110	20	87	360	0	46.01	49.68	29.77	0.28
2.5	110	5	87	360	6	28.46	4.35	22.68	1.32
2.5	110	20	87	360	6	44.32	47.97	27.34	0.22
2.5	100	10	63	330	0	19.17	33.01	24.52	0.28
2.5	120	10	63	330	0	46.47	1.78	28.69	2.99
2.5	100	10	63	330	6	7.09	36.58	23.13	0.2
2.5	120	10	63	330	6	41.24	23.44	26.97	1.8
2.5	100	10	63	400	0	14.55	50.50	25.16	0.25
2.5	120	10	63	400	0	59.15	1.70	33.53	2.50
2.5	100	10	63	400	6	26.06	51.58	24.91	0.24
2.5	120	10	63	400	6	57.56	4.50	28.07	2.52
1.0	110	10	38	330	3	18.42	52.13	24.42	0.11
5.0	110	10	38	330	3	37.84	5.18	26.72	2.45
1.0	110	10	38	400	3	29.35	45.75	26.14	0.19
5.0	110	10	38	400	3	47.90	0.00	26.75	2.39
1.0	110	10	87	330	3	26.99	45.85	25.22	0.24
5.0	110	10	87	330	3	36.62	0.00	24.70	1.77
1.0	110	10	87	400	3	33.97	35.37	25.76	0.26
5.0	110	10	87	400	3	43.37	3.66	27.52	2.91
2.5	100	5	38	360	3	19.10	36.49	24.02	0.19
2.5	120	5	38	360	3	42.49	4.03	28.70	2.15
2.5	100	20	38	360	3	32.72	48.51	26.68	0.20
2.5	120	20	38	360	3	75.57	58.20	42.86	0.66
2.5	100	5	87	360	3	14.61	6.72	23.03	0.43
2.5	120	5	87	360	3	47.95	3.93	26.91	2.50
2.5	100	20	87	360	3	27.75	47.49	25.47	0.19
2.5	120	20	87	360	3	54.14	55.60	31.31	0.30
2.5	110	10	63	360	3	31.81	43.88	26.43	0.31
2.5	110	10	63	360	3	32.54	44.48	26.43	0.31
2.5	110	10	63	360	3	32.84	44.73	26.43	0.31
2.5	110	10	63	360	3	26.66	38.25	26.67	0.52
2.5	110	10	63	360	3	33.99	45.96	29.14	0.35

**Table 6:** Chemical composition of the desilicated bauxite (DB) obtained at the optimal conditions (Al_2_O_3_ concentration of 150 g L^–1^, Na_2_O = 330 g L^–1^ used for desilication at 100 °C and L:S ratio = 20, τ = 1 h).

Main Components, wt. %										
Al_2_O_3_	Fe_2_O_3_	SiO_2_	CaO	TiO_2_	CO_2_	Na_2_O	MnO	MgO	K_2_O	LOI
47.61	34.79	2.23	1.45	2.39	0.67	0.19	0.58	0.54	0.12	9.44

LOI—lost on ignition.

**Table 7:** Experimental matrix and results obtained for the extraction of Al from the desilicated bauxite.

Exp. No.	Time (min)	Temperature (°C)	r_0_ (μm)	Al Extraction (%)
1	10	220	38	58.00
2	30	220	38	76.82
3	40	220	38	86.07
4	60	220	38	94.04
5	22.5	220	38	74.20
6	40	180	38	65.80
7	60	180	38	77.00
8	15	140	38	36.70
9	60	140	38	55.79
10	10	220	78	57.30
11	30	220	78	68.20
12	60	220	78	77.80
13	30	220	63	79.62
14	10	220	63	66.50
15	60	220	63	92.52

**Table 8:** Chemical composition of the solid residue (red mud) obtained by the leaching of desilicated bauxite by the mother aluminate solution at T = 220 °C, τ = 120 min, C_Na2O_ = 330 g L^−1^, and C_Al2O3_ = 150 g L^−1^.

Main Components, wt. %													
Fe (Total)	Fe(II)	Ti	Al	Si	Ca	Mg	Na	Mn	C	S	P	Sc	LOI
60.65	20.00	3.96	1.41	0.75	1.11	0.67	0.76	0.74	0.12	0.02	0.01	0.014	3.10

**Table 9:** Possible reactions between the raw bauxite minerals in the desilication process and following Bayer digestion in the presence of Fe^2+^ ions.

No.	Mineral	Reaction
1	Boehmite (γ-AlO(OH))	γ-AlO(OH) + NaOH + H_2_O → Na[Al(OH)_4_]
2	Hematite (Fe_2_O_3_)	Fe_2_O_3_ + HFeO_2_^−^ → Fe_3_O_4_ + OH^−^
3	Rutile (TiO_2_)	2HFeO_2_^−^+ TiO_2_ → Fe_2_TiO_4_ + 2OH^−^
4	Chamosite (Fe^2+^_3_Mg_1.5_AlFe^3+^_0.5_Si_3_AlO_12_(OH)_6_)	Fe^2+^_3_Mg_1.5_AlFe^3+^_0.5_Si_3_AlO_12_(OH)_6_ + 8NaOH → 3HFeO_2_^−^ + 0.5FeO_2_^−^ + 2Na[Al(OH)_4_] + 1.5Mg(OH)_2_ + 3Na_2_SiO_3_ + H_2_O
5	Quartz (SiO_2_)	SiO_2_ + NaOH → Na_2_SiO_3_
6	Diaspore (α-AlO(OH))	α-AlO(OH) + NaOH + H_2_O = Na[Al(OH)_4_]

## Data Availability

All data are presented in this article.
